# A Rare Case Report of NEHI in a Preterm Infant with Review of the Literature

**DOI:** 10.1155/2022/7907338

**Published:** 2022-08-12

**Authors:** Chetna Mangat, Mikaela DeCoster, Natasa Milosavljevic, Lisa Hiskey, Elizabeth H. Ristagno, Nadir Demirel

**Affiliations:** ^1^Department of Pediatrics and Adolescent Medicine, Mayo Clinic Health System, Eau Claire, WI 54701, USA; ^2^Medical College of WI- Central Wisconsin, Wausau, WI 54401, USA; ^3^Department of Pediatric Infectious Disease, Mayo Clinic Rochester, Rochester, MN 55905, USA; ^4^Department of Pediatric Pulmonology, Mayo Clinic Rochester, Rochester, MN 55905, USA

## Abstract

**Background:**

Neuroendocrine cell hyperplasia of infancy (NEHI) is a rare respiratory disorder. During infancy, it typically presents with hypoxemia, tachypnea, and respiratory distress, and is commonly misdiagnosed as common childhood illnesses such as pneumonia, reactive airway disease, or bronchiolitis. Lack of awareness about this relatively new and rare disorder in primary care and acute care settings lead to delayed diagnosis and unnecessary use of antibiotics. *Case Presentation*. We present a case of a 7-month-old girl, born prematurely at 32 weeks with tachypnea and respiratory distress who was initially diagnosed with viral pneumonia, then upper respiratory infection, and finally with community-acquired bacterial pneumonia, while the child never had any fever or upper respiratory symptoms. Failure of outpatient treatment with oral antibiotic and bronchodilator, with the persistence of respiratory symptoms such as retractions, bilateral crackles, and hypoxemia led to hospitalization for intravenous antibiotics. Given persistent symptoms, further evaluation was performed, and she was diagnosed with NEHI based on characteristic chest CT findings.

**Conclusions:**

Viral respiratory infections are the most frequent cause of respiratory illnesses in the first years of life. Primary care providers should be aware of less frequent causes of persistent respiratory symptoms in infancy like NEHI and other interstitial lung diseases in children. This may prevent unnecessary use of antibiotics and delayed diagnosis.

## 1. Introduction

Neuroendocrine cell hyperplasia of infancy (NEHI) is a type of childhood interstitial lung disease which usually presents in early infancy [1]. It was originally named persistent tachypnea of infancy but has subsequently been renamed as NEHI based on characteristic hyperplasia of pulmonary neuroendocrine cells (PNEC) on histological examinations of lung biopsies [2, 3]. NEHI typically presents in full-term infants within the first year of life with chronic respiratory symptoms such as tachypnea, hypoxemia, retractions, or respiratory crackles and is commonly associated with failure to thrive [1, 4, 5]. When suspected, chest high resolution computed tomography (HRCT) is performed, and images typically show a characteristic pattern of diffuse ground-glass opacities in the right middle lobe and lingula with varying degrees of air trapping [6–8]. Lung biopsy used to be the gold standard in diagnosis, but in most cases, a detailed clinical evaluation and HRCT are sufficient to make the diagnosis [9].

NEHI is a rare disorder of infancy, and delay in the diagnosis is not different from other rare disorders [10]. We report a case of a preterm infant with tachypnea and persistent respiratory symptoms who was diagnosed at first as upper respiratory infection, bronchiolitis, reactive airway disease, and then community-acquired pneumonia. Failure to respond to antibiotic treatment and persistence of symptoms led to a thorough diagnostic evaluation and consultation with a pediatric pulmonologist, which resulted in a diagnosis of NEHI.

## 2. Case Report

The patient was born vaginally at 32 weeks of gestational age with a birth weight of 1770 grams. Mother was induced at 32 weeks for preeclampsia and gestational diabetes. The patient had respiratory distress at birth and required CPAP for 14 hours before transitioning to room air. The patient required NG feeding for the first several weeks but successfully transitioned to full oral feeding. The newborn screen was negative.

At her 6-month well-child visit, the mother expressed concern about an increased respiratory rate. The primary care provider noted fine crackles on chest auscultation. A chest X-ray (CXR) revealed mild hyperinflation and bilateral perihilar interstitial scattered patchy pulmonary opacities. The patient was not febrile and did not seem to be in respiratory distress. She was diagnosed with viral pneumonia; supportive care was recommended.

Three weeks later, the patient was evaluated in urgent care for tachypnea and increased work of breathing. She did not have any signs of upper respiratory illness or fever.

On physical examination, she had tachypnea, nasal flaring, and intercostal retractions and oxygen saturation was 94% on room air. On auscultation, wheezing was noted with fine crackles bilaterally. Wheezing improved after albuterol nebulization, but crackles persisted bilaterally. She tested negative for Respiratory Syncytial Virus and Influenza A&B. CXR revealed mild patchy airspace opacities in the lung base, more pronounced on the right side, and mild peribronchial thickening. She was diagnosed with community-acquired bacterial pneumonia, but reactive airway disease could not be ruled out. She was discharged home on amoxicillin and albuterol inhaler as needed. Her symptoms did not improve, and she continued to have tachypnea, and intermittent shortness of breath was seen in the emergency room, where she was given oral dexamethasone and was discharged home; continuation of albuterol and amoxicillin was recommended.

A day later, she had a follow-up in the primary pediatrician's clinic and she was admitted for persistent of respiratory symptoms with oxygen saturation of 91% on room air. A repeat CXR did not reveal any significant changes from the last *X*-ray. CBC and CRP were unremarkable, but she was started on intravenous (IV) ceftriaxone and oral azithromycin therapy for presumed bacterial pneumonia. Additionally, she was started on oral prednisolone for likely acute exacerbation of reactive airway disease. Interestingly, during the time of her illness, she never spiked fevers, nor did she have any upper respiratory symptoms such as runny nose, nasal congestion, or coughing spells. She required supplemental oxygen for tachypnea and intermittent suprasternal and intercostal retractions. An echocardiogram was obtained, and there were no signs of congestive heart failure or congenital heart anomalies. Albuterol nebulization treatments did not improve her respiratory distress.

The patient was transferred to a tertiary children's hospital. There, she was evaluated by a pediatric pulmonologist and an infectious disease specialist. She continued to be afebrile. CBC and CRP were again normal, and antibiotics were discontinued due to low suspicion for bacterial infection. Respiratory panel from nasopharyngeal swab was negative for Adenovirus, Coronavirus 229E, Coronavirus HKU1, Coronavirus NL63, Coronavirus OC43, Human Metapneumovirus, Human Rhinovirus/Enterovirus, Parainfluenza virus 1–4, RSV, Bordetella pertussis, Chlamydophila *pneumoniae*, Mycoplasma *pneumoniae*, Influenza A and Influenza B. Bronchoscopy did not demonstrate any anatomic or structural abnormalities. Bronchoalveolar lavage fluid had 81% alveolar macrophages, 2% lymphocytes, 7% neutrophils. 5% eosinophils, and 5% other lining cells. Gram stain and fungal smear/culture were negative. Mycoplasma PCR, acid-fast smear/mycobacterial culture and bacterial culture, and respiratory pathogen panel were also negative. Pneumocystis carinii (PCP) PCR resulted as positive; however, PCP smear was negative. The crossing point for the PCR was 35.76, which is quite high, indicating a very low organism burden. Given that the PCP smear was also negative, it was felt that the positive PCP PCR likely represented colonization. As PCP is typically only seen in the context of severe immunosuppression, a comprehensive immunologic workup was performed. T and B cell subsets were normal with a very robust CD4 count of 3,586, and negative HIV Ab/Ag screen, immunoglobulin G, A, and E were normal, and IgM was mildly low for age. CBC with differential and LDH levels were within normal limits; she also had a video swallow study with no laryngeal penetration or aspiration identified.

**Figure 1:**
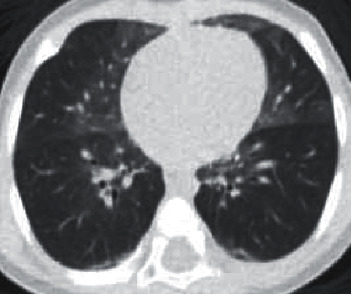
CT chest axial view shows groundglass opacities primarily involving right middle lobe and lingula.

**Figure 2:**
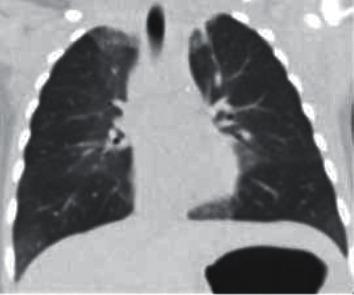
CT chest coronal view shows patchy central groundglass opacities and lower lobe atelectasis.

Chest HRCT without IV contrast demonstrated the characteristic findings of NEHI which includes central predominant ground-glass opacities involving primarily the right middle lobe and lingula, as well as the medial upper and lower lobes to a lesser degree (Figure 1 and Figure 2). Linear opacities were also seen in the left apex medially and the bibasilar lower lobes, consistent with atelectasis.

CT Images in Figures 1 and 2 show ground-glass opacities in the right middle lobe, lingula, and atelectasis of the lower lobes.

A comprehensive genetic test for pulmonary diseases was performed. The PulmoGene Panel contains 64 genes that are associated with genetic conditions that have a pulmonary component, such as cystic lung disease, bronchiectasis, idiopathic pulmonary fibrosis, and pulmonary hypertension. It includes testing for several syndromes: Alpha-1-antitrypsin.

Deficiency, Birt-Hogg-Dube syndrome, congenital central hypoventilation syndrome, cutis laxa and emphysema, cystic fibrosis, hereditary hemorrhagic telangiectasia, Hermansky- Pudlak syndrome, primary ciliary dyskinesia, and tuberous sclerosis complex associated lymphangiomyomatosis [10]. A heterozygous variant was identified in *DNAH* [11] gene which is associated with autosomal recessive primary ciliary dyskinesia.

The patient was discharged home on supplemental oxygen 1/2 per minute via nasal cannula. Given her underlying lung disease, need for chronic oxygen therapy, and underlying prematurity, the patient received palivizumab prophylaxis for the following winter season.

The patient is currently 34 months old and continues to require intermittent oxygen supplementation. Her growth percentile is between the 3^rd^ to 5^th^ percentile but stable. The patient is followed closely by nutrition and pulmonology.

## 3. Discussion

NEHI is a rare type of early childhood interstitial lung disease and the incidence and prevalence remain unknown. NEHI term was first coined in 2005 and now it is classified as a type of children's interstitial and diffuse lung disease (chILD) [1, 3]. The typical presentation is between 3 and 7 months of age with insidious onset of chronic respiratory symptoms in otherwise healthy children. The most prominent clinical features are tachypnea, hypoxemia, and crackles in lower lung fields. [2, 4, 5] Wheezing and cough are less common symptoms reported in NEHI cases but failure to thrive is commonly associated with NEHI. [5] Delay in diagnosis is common as children undergo evaluation focused on more common pulmonary conditions like upper and lower respiratory infections and other common chronic lung diseases. Awareness of rare disorders such as NEHI continues to be an issue and is the likely cause behind delayed diagnosis and unnecessary use of antibiotics and steroids in these cases [12, 13].

Historically, lung biopsy was often performed in addition to HRCT to confirm a diagnosis of NEHI. [3] Hyperplasia of PNEC and increased level of bombesin are pathognomic features on histological examinations of lung tissues in NEHI [2, 3]. However, a study conducted by Brody, et al. in 2010 looked at 23 CT scans of children with biopsy-proven NEHI and reported a sensitivity of 78% and a specificity of 100% in the diagnosis of NEHI [7], suggesting that lung biopsy is not necessary for the diagnosis of NEHI if there are specific HRCT findings with typical clinical presentation and symptomatology [6, 7]. Currently, the histopathological exam is not performed routinely to confirm the diagnosis due to the distinctive ground-glass appearance on HRCT. This unique pattern of ground-glass opacities in the right middle lobe and lingula with varying degrees of air trapping is characteristic of NEHI. [8]. Rauch et al. examined 89 cases of NEHI retrospectively and concluded that NEHI can be diagnosed based on detailed, clinical findings, and HRCT and lung biopsy may be limited to rare, complicated cases which could reduce the need for an invasive procedure that could be potentially harmful [9]. NEHI clinical score was recently proposed by Liptzin et al. for clinical evaluation of NEHI and the authors also recommended that comorbidities such as gastroesophageal reflux, aspiration, and immune system abnormalities should also be evaluated at the time of initial evaluation as they may influence clinical course and treatment [14].

Proposed factors in the pathogenesis of NEHI include abnormalities in acute or chronic oxygen sensor function of PNEC, dysregulation of neurogenic gene expression, and the resultant effects of cytokines derived from low-grade airway inflammation [15]. Bush et al. proposed that rather than being a discrete condition, NEHI could be the result of airway dysmaturation, which may co-exist with other maturational lung defects [16]. Additionally, two studies have proposed possible genetic mechanisms for NEHI. One study identified four families containing nine patients with a NEHI diagnosis suggesting a genetic connection [17]. Another study reported a mutation in *NKX2-1* in a NEHI patient with a family history of various lung diseases [18]. However, the *NKX2-1* mutation may result in but is not the predominant cause of NEHI. Thus, the exact etiology of NEHI remains unclear.

There is no specific treatment for NEHI, but most of the patients require prolonged supplemental oxygen through the second year of life [14, 19]. In our case, the patient currently is 34-month-old and continues to require supplemental oxygen at night. Generally, the outcome is good with no oxygen requirements and normal exercise tolerance later in life. Nevel et al. proposed that long-term impact on lung functions and associated lifelong complications is possible in some familial form of NEHI [18]. However, the long-term prognosis of NEHI is unknown. In our case, the presence of prematurity was a confounder. NEHI in the setting of prematurity is very rare in the literature [14, 19].

## 4. Conclusions

Tachypnea, hypoxemia, and respiratory distress in infants are commonly associated with viral bronchiolitis or pneumonia. However, if there is clinical-diagnostic discordance and persistence of symptoms, other uncommon etiologies such as NEHI should be considered. While more research is needed regarding the exact etiology of NEHI, it is important that primary care clinicians are aware of the symptoms and clinical presentation of NEHI. This case report of NEHI may help educate primary care providers about NEHI so that timely diagnosis could prevent unnecessary use of antibiotics and excessive resource use.

## Data Availability

The authors confirm that the data supporting the findings of this study are available within the article.
